# Lymphotoxin-alpha polymorphisms and presence of cancer in 1,536 consecutive autopsy cases

**DOI:** 10.1186/1471-2407-8-235

**Published:** 2008-08-13

**Authors:** Kako Takei, Shinobu Ikeda, Tomio Arai, Noriko Tanaka, Masaaki Muramatsu, Motoji Sawabe

**Affiliations:** 1 Department of Molecular Epidemiology, Medical Research Institute, Tokyo Medical and Dental University, Tokyo, 101-0062, Japan; 2 Department of Pathology, Tokyo Metropolitan Geriatric Hospital, Tokyo, 173-0015, Japan; 3 Department of Biostatistics, Harvard School of Public Health, Boston, MA, 02115, USA

## Abstract

**Background:**

Lymphotoxin-alpha (LTA) is a pro-inflammatory cytokine with anti-tumor activity. The objective of this study was to determine whether *LTA *polymorphisms influence the presence of cancer.

**Methods:**

*LTA *polymorphisms C804A (rs1041981, T60N) and T495C (rs2229094, C13R) were determined in 1,536 consecutive autopsy cases and were registered in the Japanese single-nucleotide polymorphisms (SNPs) for geriatric research (JG-SNP) Internet database. Tumors were systematically reviewed, pathologically confirmed, and assessed in relation to *LTA *genotype.

**Results:**

The study population consisted of 827 males and 709 females, with a mean age of 80 years. Altogether, we studied 606 subjects without cancer and 930 subjects with cancer of the stomach (n = 183), lung (n = 164), colon or rectum (n = 143), or other sites. The presence of cancer was higher in males than in females. The C804A and T495C polymorphisms were associated with cancer in males (CA + AA: CC, adjusted OR = 0.72, 95% CI = 0.53 – 0.99; TC + CC: TT, adjusted OR = 1.45, 95% CI = 1.04 – 2.02; respectively) but not in females. In males, the C804A polymorphism was associated with lung cancer (CA + AA: CC, adjusted OR = 0.60, 95% CI = 0.37 – 0.97), whereas the T495C polymorphism was associated with gastric cancer (TC + CC: TT, adjusted OR = 1.68, 95% CI = 1.06 – 2.65).

**Conclusion:**

We found some evidence of an association between *LTA *polymorphisms and cancer risk in elderly Japanese men. Further studies in larger populations should examine this hypothesis.

## Background

Elderly people often experience chronic and low-grade inflammation, whereupon the levels of certain inflammatory serum markers (e.g., C-reactive protein) and pro-inflammatory cytokines (e.g., tumor necrosis factor-alpha and interleukin-6) are elevated. However, increases in circulating inflammatory markers are often not noticeable in healthy elderly persons and are much below the levels experienced during acute infections [1,2]. Moreover, it is well known that the prevalence of cancer increases sharply with age, and that the majority of cancer cases occur in patients over the age of 65 [3]. Persistent inflammation is thought to trigger certain cancers, particularly those of the stomach, colon, and lung [4]. Although it has been established that the risks of cancer and inflammation increase with age [2], the influence of inflammatory cytokine polymorphisms on the genetic predisposition to cancer remains unclear.

Lymphotoxin-alpha (LTA), a member of the tumor necrosis factor (TNF) family of cytokines, was initially isolated on the basis of an anti-tumor activity. Later, this cytokine was shown to have inflammatory and immunologic activities [5]. The *LTA *gene is located within the class III region of the major histocompatibility complex (MHC) in chromosome 6p21.3 [6]. LTA plays a key role in communication between lymphocytes and stromal cells, thereby eliciting cytotoxic effects on cancer cells [7,8]. LTA induces the expression of vascular cell-adhesion molecule 1 (VCAM1) on vascular endothelial cells and recruits natural killer (NK) cells to parenchymal organs and tumor lesions [9]. NK cells have nonspecific host-defense mechanisms that aid in tumor rejection and protection from metastases. Previous studies have shown that tumor growth and metastasis are enhanced in *LTA*-deficient mice, which produce NK cells that have reduced anti-tumor potential [10,11]. Thus, LTA signaling plays an important role in anti-tumor surveillance via the maturation and recruitment of NK cells.

Previous studies have examined the relationship between *LTA *polymorphisms and various cancers, and found that the *NcoI *restriction fragment length polymorphism (A252G) in the first intron is in tight linkage disequilibrium with C804A, resulting in the substitution of threonine with asparagine at codon 60 in exon 3 [12,13]. The *LTA *252G allele increases LTA at the levels of mRNA and protein [12]. Previous studies have found that the relationship between the risk of cancer and the rate of survival among those with the *LTA *polymorphism varies according to the cancer type [14-17]. However, the effect of *LTA *polymorphisms on the presence of cancer in general is not known.

Toward this end, we searched for two non-synonymous polymorphisms, C804A (rs1041981, T60N) and T495C (rs2229094, C13R), in consecutive autopsy cases and determined whether they influenced cancer presence.

## Methods

### Subjects

The study population consisted of 1,536 consecutive autopsy cases performed at the Tokyo Metropolitan Geriatric Hospital between 1995 and 2004. *LTA *polymorphisms were registered in the Internet database of Japanese single-nucleotide polymorphisms (SNPs) for geriatric research (JG-SNP) [18]. Subjects were enrolled regardless of the cause of death and autopsies were performed on 40% of patients who died at the hospital. The study population included subjects who died from malignant disease (33%), coronary heart disease (20%), and pneumonia (13%). These proportions were similar to the causes of death reported in a survey conducted by the Ministry of Health, Labor and Welfare of Japan  which found that 30% of the Japanese population died from malignant disease, 16% from coronary heart disease, and 10% from pneumonia. The presence, absence, and histopathology of cancers were determined from autopsy findings. Histories of smoking and alcohol drinking status were retrospectively determined from medical records, and subjects were classified as smokers (including ex-smokers) versus non-smokers, and alcohol drinkers versus non-drinkers. Information on smoking and alcohol drinking status was missing from 109 and 118 patient histories, respectively. Written informed consent was obtained from the family of each patient at the time of autopsy. The study protocol was approved by the ethical Committees of the Tokyo Metropolitan Geriatric Hospital and the Tokyo Medical and Dental University.

### Genotyping

Genomic DNA was extracted from the renal cortex using a standard procedure. *LTA *polymorphisms were genotyped via melting curve analysis [19]. PCR primers and probes were as follows. For *LTA *C804A (rs1041981, T60N): 5'-GTT GGC CTC ACA CCT TCA-3' (forward primer), 5'-TGG ATG CTT GGG TTC CTG-3' (reverse primer), CAG CAC CCT CAA ACC TGC-Fluorescein (anchor probe), and LC Red 640-GCT CAC CTC ATT GGT AAA CAT CCA CCT GAC CTC C-Phosphate (detection probe). For *LTA *T495C (rs2229094, C13R): 5'-CTC TTT CTC TGC AGG TTC TC-3' (forward primer), 5'-GCT CTA GGG CTC AAG GTT T-3' (reverse primer), CTC CCA AGG GTG GGT G-Fluorescein (anchor probe), and LCRed640-CAC CAC CCT ACA CCT CCT CCT TCT GG-Phosphate (detection probe). The PCR amplification reaction was performed in 5 μl volume using the LightCycler PCR kit (Roche Diagnostics, Penzberg, Germany). Each well contained 1 × PCR buffer, 4 mM of MgCl2, 0.2 mM of dNTPs, 0.05 μM of forward primer, 0.5 μM of reverse primer, 0.2 μM of each anchor and detection probe, 0.1 U of Faststart Taq polymerase, and 10 ng of genomic DNA. The cycling protocol was performed as follows: initial denaturation at 94°C for 10 min; followed by 40 cycles of 94°C for 15 sec, 55°C for 15 sec, and 72°C for 15 sec; and a final extension step at 72°C for 10 min. After completion of PCR, the plates were heated from 40°C to 90°C with a gradient of 0.1°C per second immediately before melting temperature analysis on the LightCycler 480 Instrument (Roche Diagnostics). The genotype was determined based on melting profiles automatically classified by LightCycler Genotyping software (Roche Diagnostics). C804A and T495C polymorphisms were genotyped with success rates of 96% and 95%, respectively. Genotyping accuracy was confirmed by sequencing randomly selected samples. The melting temperature analysis correlated entirely with the direct sequencing results.

### Statistical analysis

Statistical analysis was performed using SAS software, version 9.1.3 (SAS Institute Inc., Cary, NC). The distribution of subjects with and without cancer, and associations with other variables, were compared using Fisher's exact test. Hardy-Weinberg equilibrium (HWE) was assessed using a permutation test. Odds ratio (OR) and 95% confidence interval (CI) were calculated using an unconditional logistic regression model that compared genotype frequencies in subjects with and without cancer, with respect to *LTA *polymorphisms. The OR was adjusted by age, sex, smoking, and alcohol drinking status. This study had a statistical power of 0.8 to detect an OR of 1.5 in carriers of at least one polymorphic allele, compared with carriers of homozygous wild-type alleles. *LTA *haplotype frequencies were estimated using Haploview software . Chi-square values were calculated to determine the distribution of *LTA *haplotypes among subjects with and without cancer. A probability level of 5% was considered statistically significant for all analyses.

## Results

### Characteristics of the study subjects

Selected demographic variables, including the sites and histological types of cancers, and risk factors are shown in Table 1 (Additional file 1). The mean age of subjects was 80.2 ± 8.9 years. The study population consisted of 827 (54%) males and 709 (46%) females. Altogether, we studied 606 subjects without cancer and 930 subjects with cancer. When the total number of subjects was divided by the number of subjects over or under the median age of 80 years, the distributions of subjects with or without cancer did not significantly differ. The frequency of cancer was significantly higher in males than in females. No differences in smoking and alcohol drinking status between cancer-bearing and cancer-free subjects were observed. The most frequent sites of cancer were the stomach (n = 183), lung (n = 164), and colon or rectum (n = 143).

### Genotype and allele frequencies of *LTA *polymorphisms

C804A and T495C were genotyped in all subjects. The genotype frequencies of C804A were 37% for CC, 48% for CA, and 15% for AA. The minor allele frequency was 39%, and the allele distribution was consistent with HWE (p = 0.65). The genotype frequencies of T495C were 66% for TT, 31% for TC, and 3% for CC. The minor allele frequency was 19% and the allele distribution was consistent with HWE (p = 0.48).

### Association of *LTA *polymorphisms with cancer overall

The associations between C804A and T495C polymorphisms and the presence of cancer are shown in Table 2 (Additional file 1). Among cancer-free subjects, the frequencies of the C804A genotypes CC, CA, and AA were 33%, 49%, and 18%, respectively, in males, and 33%, 54%, and 13%, respectively, in females (Table 3) (Additional file 1). In comparison with the CC genotype, the CA genotype was associated with a significantly lower presence of cancer in all subjects (adjusted OR = 0.78, 95% CI = 0.61 – 0.99). In males, the CA + AA genotype was associated with a significantly lower presence of cancer compared with the CC genotype (adjusted OR = 0.72, 95% CI = 0.53 – 0.99). The association between the C804A polymorphism and cancer in females was not significant (CA + AA: CC, adjusted OR = 0.92, 95% CI = 0.66 – 1.29). Additionally, cancer frequency was not associated with C804A (data not shown).

In cancer-free subjects, the frequency of the T495C genotypes TT, TC, and CC were 70%, 28%, and 2%, respectively, in males, and 66%, 31%, and 3%, respectively, in females (Table 3 in Additional file 1). Compared with the TT genotype, the CC genotype was associated with a significantly higher presence of cancer (adjusted OR = 2.24, 95% CI = 1.09 – 4.61). In males, the TC + CC genotype was associated with a significantly higher presence of cancer, compared with the TT genotype (adjusted OR = 1.45, 95% CI = 1.04 – 2.02). The association between the T495C polymorphism and cancer in females was not significant (TC + CC: TT, adjusted OR = 1.08, 95% CI = 0.77 – 1.50).

Additionally, we explored the relationship between the presence of C804A and T495C haplotypes and tumor formation. C804A and T495C appeared to be present in moderate linkage disequilibrium (r^2 ^= 0.15, D' = 1.0) and three major haplotypes were identified (495T-804C, 495T-804A, and 495C-804C). None of these haplotypes were associated with the presence of cancer (data not shown).

### Association of *LTA *polymorphisms with specific types of cancers

Significant associations between *LTA *polymorphisms and the presence of cancer in males prompted us to explore the relationships between these polymorphisms and the sites of cancer. After analyzing subjects with stomach, lung, colorectal, prostate, breast, liver, biliary tract, kidney or urinary tract, and hematopoietic malignancies, we observed a positive association between *LTA *polymorphisms and lung and stomach cancers (Table 4 in Additional file 1). Among male subjects with lung cancer, the C804A CA + AA genotype was associated with a significantly lower presence of cancer than was the CC genotype (adjusted OR = 0.60, 95% CI = 0.37–0.97). The association between C804A polymorphism and lung cancer in females was not significant (CA + AA: CC, adjusted OR = 0.65, 95% CI = 0.35 – 1.23).

The T495C polymorphism was not associated with the presence of lung cancer, but the TC + CC genotype was associated with a significantly higher presence of gastric cancer in males, compared with the TT genotype (adjusted OR = 1.68, 95% CI = 1.06–2.65). The association between T495C polymorphism and gastric cancer was not significant in females (TC + CC: TT, adjusted OR = 1.16, 95% CI = 0.61 – 2.21).

## Discussion

Our findings demonstrate that the *LTA *polymorphism C804A is associated with a lower presence of cancer, particularly lung cancer, in elderly Japanese men, consistent with previous studies showing that LTA has anti-tumor activity.

Several laboratories have recently published a functional analysis of the *LTA *C804A (T60N). Compared with the C allele, the A allele is more bioactive with regards to the induction of VCAM1 in cultured human coronary-artery smooth muscle cells [13]. Furthermore, C804A is present in very high linkage disequilibrium with another polymorphism, *LTA *A252G. These two polymorphisms have been shown to be completely concordant in Japanese subjects [16,17]. Compared with the A allele, the G allele of the A252G genotype confers higher transcriptional activity in Jurkat cells [13] and phytohemagglutinin-stimulated peripheral blood mononuclear cells [12]. Thus, the C and A alleles are considered low and high bioactive alleles in *LTA*, respectively. Accordingly, the data suggest that the lower presence of cancer in our study population may reflect enhanced LTA activity because of the presence of high-bioactive alleles.

The C804A and A252G polymorphisms have been studied with regards to cancer survival rates and risks of developing various type of cancer, including lung [14], stomach [15,20,21], colorectal [22], breast [23], cervix [16], endometrium [17] and bladder [24] cancers, as well as leukemia [25], lymphoma [26,27], and myeloma [28]. The high-bioactive genotype was associated with the risk of developing cancers of the lung [14], colon or rectum [22], non-Hodgkin lymphoma [26,27], and myeloma [28]. Low-bioactive genotypes were associated with the risk of developing cervical [16] and endometrial cancers [17], but not gastric [15,20,21], breast [23], bladder cancers [24], or leukemia [25]. These differences may be partially explained by the multi-functionality of LTA. LTA can promote cell growth and adhesion, and can potentially favor the growth of certain tumors.

It remains unclear why C804A is associated with cancer in males but not in females. However, gender is known to play an important role in the development of various cancers. Recent studies also suggest that cytokine secretion and innate immunity differ between the genders [29,30].

The C13R (T495C) polymorphism has not been as thoroughly studied as the T60N (C804A) polymorphism. Previous study reported there was not an association between the T495C polymorphism and lung cancer [31]. The T495C polymorphism is a haplotype component associated with altered LTA expression and increased levels of vascular- and autoimmune-mediated inflammation [32]. These findings suggest that the T495C polymorphism is also associated with cancer. We observed an association between the C allele of the T495C polymorphism and the presence of cancer, particularly gastric cancer. However, as the minor allele frequency for this polymorphism was low, these results must be confirmed in a larger study population.

The *LTA *gene is located within the class III region of the MHC, in the 6p21.3 chromosome [6]. This region contains many other genes, including that encoding the pro-inflammatory cytokine, TNF. It is well-known that this region shows high degrees of linkage disequilibrium. Thus, associations identified in this study may reflect the effects of other gene variants in this region.

As this was a hospital-based autopsy study, we had limited access to information on lifestyle variables that could potentially influence the development of cancer. Furthermore, although autopsies were performed on many of the deaths (40%) that occurred in the hospital, and the causes of death among our autopsy cases were similar to those reported in a national survey, we cannot rule out the possibility of selection bias. Such bias can arise from chance of admission, consent to autopsy, cause of death, and autopsy practice. Survival bias is also a possibility, as particular genotypes may be associated with other diseases or influence the lifespan of certain subjects, thereby introducing bias into the study population. However, the C804A genotype frequency was similar to that reported in other Japanese population studies [16,17], and any effects of such possible bias did not cause our results to deviate from HWE.

## Conclusion

We found some evidence of an association between *LTA *polymorphisms and cancer risk in elderly Japanese men. Further studies in larger populations should examine this hypothesis.

## Competing interests

The authors declare that they have no competing interests.

## Authors' contributions

KT performed the statistical analysis and drafted the manuscript. SI participated in the design and performed the statistical analysis. TA performed the pathological analysis. NT assessed the data integrity. MM coordinated the study and helped draft the manuscript. MS performed the pathological analysis and helped coordinate the study. All authors read and approved the final manuscript.

## Pre-publication history

The pre-publication history for this paper can be accessed here:



## Supplementary Material

Additional file 1 Tables 1–4. Table 1 – Distribution of selected demographic variables and risk factors. Table 2 – Associations between *LTA *polymorphisms and overall cancer. Table 3 – Distribution of *LTA *genotypes in cancer-free and cancer-bearing subjects, lung and gastric cancers. Table 4 – Associations of *LTA *polymorphisms with lung cancer and gastric cancer. Click here for file
