# Supplementary material for: Lymphotoxin-alpha polymorphisms and presence of cancer in 1,536 consecutive autopsy cases
Source: BMC Cancer. 2008 Aug 13;8:235. doi: 10.1186/1471-2407-8-235 (PMC2527017; doi:10.1186/1471-2407-8-235)
Supplement: Additional file 1 — Tables 1–4. Table 1 – Distribution of selected demographic variables and risk factors. Table 2 – Associations between LTA polymorphisms and overall cancer. Table 3 – Distribution of LTA genotypes in cancer-free and cancer-bearing subjects, lung and gastric cancers. Table 4 – Associations of LTA polymorphisms with lung cancer and gastric cancer. [file 1471-2407-8-235-S1.doc]

| **Table 1. Distribution of selected demographic variables and risk factors.** | | | | | | | |
| --- | --- | --- | --- | --- | --- | --- | --- |
|  | All subjects (n = 1536) | | Cancer-free (n = 606) | | Cancer-bearing (n = 930) | | p-value* |
| Age at death, years† | 80.2 ± 8.9 | |  |  |  |  |  |
| ≧80, n (%) | 828 | (54) | 346 | (57) | 482 | (52) |  |
| < 80 | 708 | (46) | 260 | (43) | 448 | (48) | 0.05 |
| Gender, n (%) |  |  |  |  |  |  |  |
| Male | 827 | (54) | 297 | (49) | 530 | (57) |  |
| Female | 709 | (46) | 309 | (51) | 400 | (43) | 0.002 |
| Smoking, n (%) |  |  |  |  |  |  |  |
| Non-smoker | 697 | (45) | 282 | (46) | 415 | (45) |  |
| Current/Ex-smoker | 730 | (48) | 265 | (44) | 465 | (50) | 0.11 |
| missing | 109 | (7) | 59 | (10) | 50 | (5) |  |
| Alcohol, n (%) |  |  |  |  |  |  |  |
| Non-drinker | 908 | (59) | 369 | (61) | 539 | (58) |  |
| Drinker | 510 | (33) | 180 | (30) | 330 | (35) | 0.05 |
| missing | 118 | (8) | 57 | (9) | 61 | (7) |  |
| Cancer, n (%) |  |  |  |  |  |  |  |
| 0 |  |  |  |  | 606 | (40) |  |
| 1 |  |  |  |  | 696 | (45) |  |
| 2 |  |  |  |  | 183 | (12) |  |
| ≧3 |  |  |  |  | 51 | (3) |  |
| Cancer sites, n |  |  |  |  |  |  |  |
| Stomach |  |  |  |  | 183 |  |  |
| Lung |  |  |  |  | 164 |  |  |
| Colorectal |  |  |  |  | 143 |  |  |
| Leukemia |  |  |  |  | 135 |  |  |
| Prostate |  |  |  |  | 96 |  |  |
| Malignant Lymphoma |  |  |  |  | 75 |  |  |
| Breast |  |  |  |  | 70 |  |  |
| Liver |  |  |  |  | 53 |  |  |
| Pancreas |  |  |  |  | 52 |  |  |
| Biliary |  |  |  |  | 52 |  |  |
| Renal, Urinary tract |  |  |  |  | 52 |  |  |
| † means ± SDs |  |  |  |  |  |  |  |
| * p value for Fisher’s exact probability test | | |  |  |  |  |  |

# Tables

| **Table 2. Associations between *LTA* polymorphisms and cancer overall.** | | | | | | | | | | | | | | | | | | | | | | | | | | | | | | | | | | | | | |
| --- | --- | --- | --- | --- | --- | --- | --- | --- | --- | --- | --- | --- | --- | --- | --- | --- | --- | --- | --- | --- | --- | --- | --- | --- | --- | --- | --- | --- | --- | --- | --- | --- | --- | --- | --- | --- | --- |
|  | | All subjects | | | | | | | | | | |  | Male | | | | | | | | | | |  | | Female | | | | | | | | | | |
|  | | Crude OR (95% CI) | | | | | Adjusted OR (95% CI)† | | | | | |  | Crude OR (95% CI) | | | | | | Adjusted OR (95% CI)‡ | | | | |  | | Crude OR (95% CI) | | | | | Adjusted OR (95% CI)‡ | | | | | |
| *LTA*, C804A (T60N) | | | | | | |  | |  | | | |  |  | | |  | | |  | |  | | |  | |  | |  | | |  | |  | | | |
| CC | | 1.00 | (reference) | | | | 1.00 | | (reference) | | | |  | 1.00 | | | (reference) | | | 1.00 | | (reference) | | |  | | 1.00 | | (reference) | | | 1.00 | | (reference) | | | |
| CA | | 0.76 | (0.61 – 0.96) | | | | 0.78 | | (0.61 – 0.99) | | | |  | 0.75 | | | (0.55 – 1.03) | | | 0.72 | | (0.51 – 1.00) | | |  | | 0.79 | | (0.57 – 1.10) | | | 0.85 | | (0.60 – 1.20) | | | |
| AA | | 0.85 | (0.62 – 1.17) | | | | 0.94 | | (0.67 – 1.32) | | | |  | 0.70 | | | (0.46 – 1.08) | | | 0.73 | | (0.47 – 1.15) | | |  | | 1.07 | | (0.67 – 1.72) | | | 1.28 | | (0.76 – 2.15) | | | |
| CA + AA | | 0.78 | (0.63 – 0.97) | | | | 0.81 | | (0.65 – 1.02) | | | |  | 0.74 | | | (0.55 – 1.00) | | | 0.72 | | (0.53 – 0.99) | | |  | | 0.84 | | (0.62 – 1.16) | | | 0.92 | | (0.66 – 1.29) | | | |
| P for trend* | |  |  | | | | 0.08 | |  | | | |  |  | | |  | | | 0.05 | |  | | |  | |  | |  | | | 0.70 | |  | | | |
|  | |  |  | | | |  | |  | | | |  |  | | |  | | |  | |  | | |  | |  | |  | | |  | |  | | | |
| *LTA*, T495C (C13R) | | | | | | |  | |  | | | |  |  | | |  | | |  | |  | | |  | |  | |  | | |  | |  | | | |
| TT | | 1.00 | (reference) | | | | 1.00 | | (reference) | | | |  | 1.00 | | | (reference) | | | 1.00 | | (reference) | | |  | | 1.00 | | (reference) | | | 1.00 | | (reference) | | | |
| TC | | 1.14 | (0.91 – 1.44) | | | | 1.18 | | (0.93 – 1.51) | | | |  | 1.20 | | | (0.87 – 1.66) | | | 1.31 | | (0.93 – 1.84) | | |  | | 1.11 | | (0.80 – 1.54) | | | 1.07 | | (0.76 – 1.51) | | | |
| CC | | 1.78 | (0.96 – 3.51) | | | | 2.24 | | (1.09 – 4.61) | | | |  | 2.92 | | | (1.10 – 7.76) | | | 4.73 | | (1.40 – 16.02) | | |  | | 1.07 | | (0.42 – 2.71) | | | 1.12 | | (0.42 – 3.03) | | | |
| TC + CC | | 1.19 | (0.96 – 1.49) | | | | 1.25 | | (0.99 – 1.58) | | | |  | 1.30 | | | (0.95 – 1.78) | | | 1.45 | | (1.04 – 2.02) | | |  | | 1.11 | | (0.81 – 1.52) | | | 1.08 | | (0.77 – 1.50) | | | |
| P for trend* | |  |  | | | | 0.09 | |  | | | |  |  | | |  | | | 0.06 | |  | | |  | |  | |  | | | 0.54 | |  | | | |
| † OR adjusted by age (divided between under 80 years and over 80 years), sex, smoking status, and alcohol habit. | | | | | | | | | | | | | | | | | | | | | | | | | | | | | | | |  | |  | | | |
| ‡ OR adjusted by age (divided between under 80 years and over 80 years), smoking status, and alcohol habit. | | | | | | | | | | | | | | | | | | | | | | | | | | | | |  | | |  | |  | | | |
| * P values for trends were calculated using a single variable for the number of variant alleles present. | | | | | | | | | | | | | | | | | | | | | | | | |  | |  | |  | | |  | |  | | | |
|  | **Table 3. Distribution of *LTA* genotypes in cancer-free and cancer-bearing subjects, lung and gastric cancers.** | | | | | | | | | | | | | | | | | | | | | | | | | | | | | | | | | | | |  |
|  |  | | |  | All subjects (n = 1536) | | | | | | | | | |  | Male (n = 827) | | | | | | | | | |  | | Female (n = 709) | | | | | | | | |  |
|  | *LTA*, C804A(T60N) | | |  | CC | | | CA | | | AA | | | |  | CC | | | CA | | | | AA | | |  | | CC | | | CA | | | | AA | |  |
|  | cancer-free | | |  | 195 | (33) | | 304 | | (52) | 89 | (15) | | |  | 96 | | (33) | 142 | | (49) | | 50 | (18) | |  | | 99 | | (33) | 162 | | (54) | | 39 | (13) |  |
|  | cancer-bearing | | |  | 358 | (39) | | 424 | | (46) | 139 | (15) | | |  | 211 | | (40) | 234 | | (45) | | 77 | (15) | |  | | 147 | | (37) | 190 | | (48) | | 62 | (15) |  |
|  | lung cancer | | |  | 73 | (45) | | 71 | | (43) | 20 | (12) | | |  | 48 | | (44) | 50 | | (46) | | 10 | (10) | |  | | 25 | | (45) | 21 | | (37) | | 10 | (18) |  |
|  | gastric cancer | | |  | 74 | (41) | | 73 | | (41) | 32 | (18) | | |  | 51 | | (40) | 50 | | (40) | | 25 | (20) | |  | | 23 | | (43) | 23 | | (43) | | 7 | (14) |  |
|  | missing | | |  | 27 |  | |  | |  |  |  | | |  | 17 | |  |  | |  | |  |  | |  | | 10 | |  |  | |  | |  |  |  |
|  |  | | |  |  |  | |  | |  |  |  | | |  |  | |  |  | |  | |  |  | |  | |  | |  |  | |  | |  |  |  |
|  | *LTA*, T495C(C13R) | | |  | TT | | | TC | | | CC | | | |  | TT | | | TC | | | | CC | | |  | | TT | | | TC | | | | CC | |  |
|  | cancer-free | | |  | 396 | (68) | | 172 | | (30) | 13 | (2) | | |  | 201 | | (70) | 79 | | (28) | | 5 | (2) | |  | | 195 | | (66) | 93 | | (31) | | 8 | (3) |  |
|  | cancer-bearing | | |  | 582 | (64) | | 289 | | (32) | 35 | (4) | | |  | 331 | | (65) | 156 | | (30) | | 24 | (5) | |  | | 251 | | (63) | 133 | | (34) | | 11 | (3) |  |
|  | lung cancer | | |  | 98 | (62) | | 52 | | (32) | 9 | (6) | | |  | 64 | | (62) | 32 | | (31) | | 7 | (7) | |  | | 34 | | (61) | 20 | | (36) | | 2 | (3) |  |
|  | gastric cancer | | |  | 110 | (62) | | 58 | | (33) | 9 | (5) | | |  | 77 | | (60) | 39 | | (30) | | 8 | (6) | |  | | 33 | | (62) | 19 | | (36) | | 1 | (2) |  |
|  | missing | | |  | 49 |  | |  | |  |  |  | | |  | 31 | |  |  | |  | |  |  | |  | | 18 | |  |  | |  | |  |  |  |

| **Table 4. Associations of *LTA* polymorphisms with lung cancer and gastric cancer.** | | | | | | | | | |
| --- | --- | --- | --- | --- | --- | --- | --- | --- | --- |
|  | Male | | | |  | Female | | | |
|  | Crude OR (95% CI) | | Adjusted OR (95% CI)† | |  | Crude OR (95% CI) | | Adjusted OR (95% CI)† | |
| Lung cancer |  |  |  |  |  |  |  |  |  |
| *LTA*, C804A (T60N) |  |  |  |  |  |  |  |  |  |
| CC | 1.00 | (reference) | 1.00 | (reference) |  | 1.00 | (reference) | 1.00 | (reference) |
| CA + AA | 0.63 | (0.40 – 0.98) | 0.60 | (0.37 – 0.97) |  | 0.61 | (0.34 – 1.09) | 0.65 | (0.35 – 1.23) |
| P for trend* |  |  | 0.02 |  |  |  |  | 0.38 |  |
| *LTA*, T495C (C13R) |  |  |  |  |  |  |  |  |  |
| TT | 1.00 | (reference) | 1.00 | (reference) |  | 1.00 | (reference) | 1.00 | (reference) |
| TC + CC | 1.46 | (0.91 – 2.34) | 1.47 | (0.89 – 2.43) |  | 1.25 | (0.69 – 2.25) | 1.04 | (0.54 – 1.98) |
| P for trend* |  |  | 0.07 |  |  |  |  | 0.48 |  |
|  |  |  |  |  |  |  |  |  |  |
| Gastric cancer |  |  |  |  |  |  |  |  |  |
| *LTA*, C804A (T60N) |  |  |  |  |  |  |  |  |  |
| CC | 1.00 | (reference) | 1.00 | (reference) |  | 1.00 | (reference) | 1.00 | (reference) |
| CA + AA | 0.74 | (0.48 – 1.13) | 0.69 | (0.44 – 1.08) |  | 0.64 | (0.36 – 1.16) | 0.69 | (0.37 – 1.31) |
| P for trend* |  |  | 0.46 |  |  |  |  | 0.26 |  |
| *LTA*, T495C (C13R) |  |  |  |  |  |  |  |  |  |
| TT | 1.00 | (reference) | 1.00 | (reference) |  | 1.00 | (reference) | 1.00 | (reference) |
| TC + CC | 1.46 | (0.94 – 2.28) | 1.68 | (1.06 – 2.65) |  | 1.17 | (0.64 – 2.14) | 1.16 | (0.61 – 2.21) |
| P for trend* |  |  | 0.06 |  |  |  |  | 0.68 |  |
| † OR adjusted by age (divided between under 80 years and over 80 years), smoking status, and alcohol habit. | | | | | | | | | |
| * P values for trends were calculated using a single variable for the number of variant alleles present. | | | | | | | | | |
